# Size-Controlled Ammonium-Based Homopolymers as Broad-Spectrum Antibacterials

**DOI:** 10.3390/antibiotics12081320

**Published:** 2023-08-16

**Authors:** Meltem Haktaniyan, Richa Sharma, Mark Bradley

**Affiliations:** 1 EaStCHEM, School of Chemistry, University of Edinburgh, Joseph Black Building, West Mains Road, Edinburgh EH9 3FJ, UK; meltemhaktaniyan@gmail.com (M.H.); cftri.ftbe.richa@gmail.com (R.S.); 2 Precision Healthcare University Research Institute, Queen Mary University of London, Whitechapel, Empire House, London E1 1HH, UK

**Keywords:** antimicrobial, bactericidal, quaternary ammonium, RAFT polymerization

## Abstract

Ammonium group containing polymers possess inherent antimicrobial properties, effectively eliminating or preventing infections caused by harmful microorganisms. Here, homopolymers based on monomers containing ammonium groups were synthesized via Reversible Addition Fragmentation Chain Transfer Polymerization (RAFT) and evaluated as potential antibacterial agents. The antimicrobial activity was evaluated against Gram-positive (*M. luteus* and *B. subtilis*) and Gram-negative bacteria (*E. coli* and *S. typhimurium*). Three polymers, poly(diallyl dimethyl ammonium chloride), poly([2-(methacryloyloxy)ethyl]trimethylammonium chloride), and poly(vinyl benzyl trimethylammonium chloride), were examined to explore the effect of molecular weight (10 kDa, 20 kDa, and 40 kDa) on their antimicrobial activity and toxicity to mammalian cells. The mechanisms of action of the polymers were investigated with dye-based assays, while Scanning Electron Microscopy (SEM) showed collapsed and fused bacterial morphologies due to the interactions between the polymers and components of the bacterial cell envelope, with some polymers proving to be bactericidal and others bacteriostatic, while being non-hemolytic. Among all the homopolymers, the most active, non-Gram-specific polymer was poly([2-(methacryloyloxy)ethyl]trimethylammonium chloride), with a molecular weight of 40 kDa, with minimum inhibitory concentrations between 16 and 64 µg/mL, showing a bactericidal mode of action mediated by disruption of the cytoplasmic membrane. This homopolymer could be useful in biomedical applications such as surface dressings and in areas such as eye infections.

## 1. Introduction

Infection and contamination caused by microorganisms has a long, global, historical precedence, with diseases such as tuberculosis (TB), leprosy, syphilis, and “plagues” causing enormous levels of death and social challenge. The reduced effectiveness of existing antibiotics due to abuse, misuse, or overuse has led to huge increases in antimicrobial resistance and is, for example, a major problem in the area of TB, with drug-resistant and extreme drug-resistant organisms. According to a systematic analysis of the global burden associated with drug-resistant infections (excluding TB), it was estimated that 1.27 million deaths were attributable to resistant bacteria (notably *E. coli*, *S. aureus*, and *K. pneumonia*) [1]. Thus, antimicrobial agents or materials that act by alternative processes and mechanisms are important in eradicating pathogenic microorganisms. Over the past few decades, antimicrobial polymers have emerged as promising agents in surface coatings [2,3,4,5] and as materials that might, in certain situations, replace existing antimicrobials, e.g., for skin infections and topical wound dressings [6,7,8]. As such, huge efforts have been made in the synthesis and application of polymers as broad-spectrum antimicrobials [9,10,11,12,13].

Bacteria are generally classified into two groups, either Gram-positive or Gram-negative, based on their distinguishable cell envelopes. Although the inner or cytoplasmic membranes of both bacteria resemble each other, the outer envelopes are highly distinctive, with a thick, crosslinked peptidoglycan surrounding the cytoplasmic membrane in Gram-positive bacteria. In Gram-negative bacteria, a thinner peptidoglycan layer, with an additional outer membrane layer containing phospholipids and lipopolysaccharides, is found [14]. As might be imagined, the interaction between cationic polymers and bacteria happens due to a variety of reasons, depending on the bacterium in question. Thus, the lipopolysaccharides within the outer layer of the cell membrane in Gram-negative bacteria are formally negatively charged at physiological pH [14,15,16] and will interact with positively charged polymers. The cell membranes of Gram-positive bacteria are likewise formally “negatively charged”, in this case due to high levels of teichoic and lipoteichoic acids [17,18]. In addition, it is well known that cationic materials bind or target specific bacterial components, such as conventional cationic antibiotics including polymyxin, which binds Lipid A [19,20], brevibacillin, which binds lipoteichoic acid [21], and nisin, which binds lipids on the cytoplasmic membranes of Gram-positive bacteria to disturb peptidoglycan synthesis [22]. As such, the rationale for cationic polymers (with their enormous diversity) being able to interact with microorganisms is strong. In addition, cationic antimicrobial polymers are chemically robust compared to conventional antibiotics [23], and their applicability in wound healing [24], contact lenses [25], and as surface coatings in biomedical devices [26,27] makes them attractive tools in the fight against pathogens.

An important class of cationic antimicrobial polymers are those that contain ammonium groups [28,29,30], which have often been added to enhance the antimicrobial activity of existing materials [31,32,33] and have been shown to impart algistatic [34], bacteriostatic or bactericidal [35], tuberculostatic [36], sporostatic [5], fungistatic [37] or fungicidal [38], and virucidal [39] activity. The antimicrobial mode of action of ammonium-group-containing polymers is not fully understood but is believed to start with the binding of the cationic groups of the polymer onto the various negatively charged bacteria cell envelope components (mentioned above) via electrostatic interactions, leading to the disorganization of its structure and the leakage of low-molecular-weight components [40,41]. The strength of the antimicrobial activity of the ammonium polymers depends on the molecular weight of the polymer [42,43], the position of the cationic center (i.e., side chain or main chain) [44], the morphology and architecture of the polymer [45], the polymer’s hydrophobic/hydrophilic balance [46,47], and perhaps the nature of the counter anion [48].

Ammonium-group-containing polymers are typically synthesized either directly from ammonium-group-bearing monomers or the quaternization of the amine groups of polymers by alkyl halides/reductive amination or protonation. Over the past few decades, controlled living polymerization techniques have enabled the fine regulation of the molecular weight distributions of synthesized polymers. One such method is Reversible Addition Fragmentation Chain Transfer Polymerization (RAFT), a powerful technique that allows the design and building of complex, versatile polymer architectures and is applicable to a wide range of monomers and solvents, including water [49]. Importantly, it does not use toxic metal salts, making it a perfect method for the synthesis of polymers for biomedical applications [50]. Antimicrobial polymers offer broad chemical scope, as they can be readily prepared from numerous monomers, with control of the molecular weights of polymers [51,52,53]. Although antibacterial polymers with ammonium groups as copolymers have been reported extensively [46,47,52], few reports have looked at the antimicrobial activity of the homopolymers of ammonium-based monomers that have been used here [36,38,54,55,56,57,58]. Among the cationic polymers, poly(2-(dimethylamino)ethyl methacrylate (PDMAEMA) is probably the most commonly used material (the amine becoming an ammonium ion upon protonation) for use in biomedical applications, with its incorporation into films, surface coatings, and synthesis via various polymerization techniques, such as Atom Transfer Radical Polymerization (ATRP), RAFT, etc. A report [36] on polymers (again cationic via physiological protonation) synthesized through RAFT polymerization, including poly(2-(dimethylamino)ethyl methacrylate) (4.5 kDa, 6.1 kDa, and 11.2 kDa), poly(2-(dimethylamino)ethyl acrylate) (11 kDa and 3.2 kDa), and poly(2-aminoethylmethacrylate) (11.2 kDa), showed antimicrobial activity against *M. smegmatis* and Gram-negative (*E. coli* and *P. putida*) bacteria. PDMAEMAs selectively killed mycobacteria over Gram-negative bacteria, while the membrane lytic activity of PDMAEMA was comparatively low, giving a good selectivity window. 

Cationic polymers can show cytotoxic effects [59] due to their interactions with the cell membrane via non-specific electrostatic interactions [60,61,62]. It has been shown that polycations possessing a higher molecular weight may exhibit greater toxicity; however, this was based upon a restricted range of polymers [63]. Amine- and guanidine-functionalized copolymers showed a correlation between the number of cationic groups and their minimum inhibitory concentration, although the degree of polymerization breadth made it complex to ascertain the effect of the larger and smaller polymers within the ensemble. Increased ratios of cationic groups can induced hemolysis [64] and an investigation to determine the cytotoxicity of PDMAEMAs (43 kDa–915 kDa) showed that the smallest polymer (43 kDa) exhibited extremely high toxicity on human brain microvascular endothelial cells [65], while the cytotoxicity and cellular membrane disruption of HepG2 cells by rhodamine B end-labeled PDMAEMAs (11–48 kDa) synthesized via ATRP showed that the shorter polymers (Mw < 17 kDa) showed reduced toxicity [60]. 

In this study, we focused on ammonium group containing homopolymers synthesized via RAFT polymerization. Specifically, we explored the impact of molecular weight on the antimicrobial activity, cytotoxicity, and biocompatibility, as well as their antimicrobial mode of action. A key advantage of these polymers is the fact that there is no need for modification, such as quaternization which typically requires alkylating agents to drive full functionalization. At the outset, five different monomers were polymerized via RAFT polymerization to give 20 kDa polymers, and the antimicrobial activity of the homopolymers was analyzed on Gram-negative (*S. typhimurium*, *E. coli*) and Gram-positive (*B. subtilis*, *M. luteus*) bacteria. Poly(diallyl dimethyl ammonium chloride) (PDADMAC) showed the best inhibition across all the bacterial strains and, due to their limited toxicity, poly([2-(methacryloyloxy)ethyl]trimethylammonium chloride) and poly(vinyl benzyl trimethylammonium chloride) were also selected, to investigate the effect of molecular weight (10, 20, and 40 kDa) on their antimicrobial activity and mechanisms of action. 

## 2. Results and Discussion

### 2.1. Synthesis and Characterization of the Homopolymers

**Figure 1:**
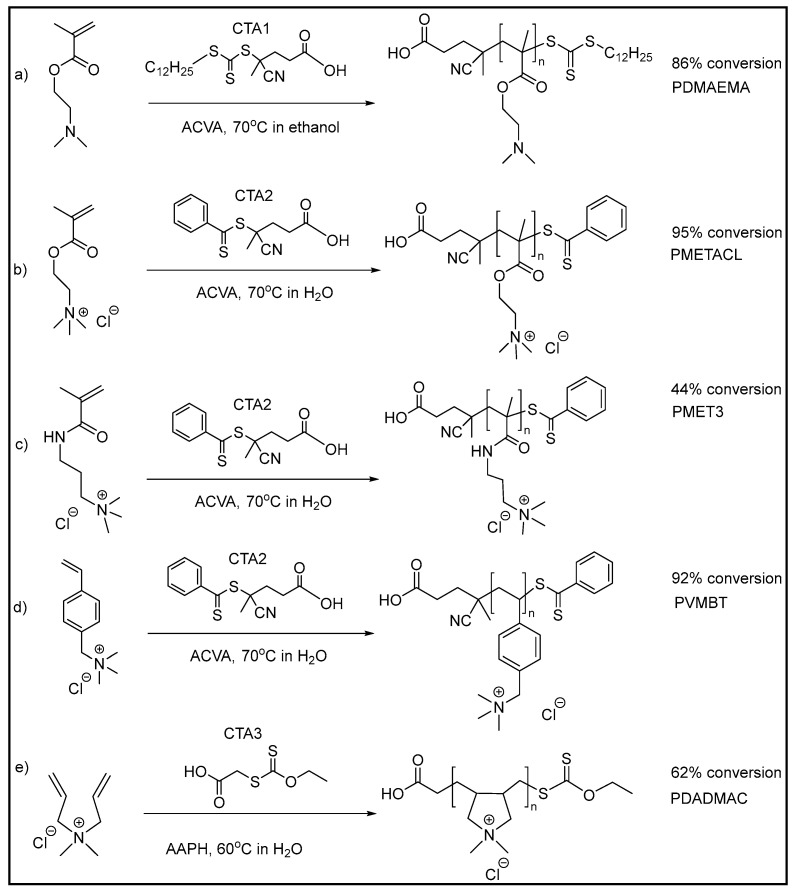
RAFT polymerization of quaternary ammonium-group-bearing monomers with final monomer conversion levels. (**a**) 2-(dimethylamino)ethyl methacrylate); (**b**) [2 (methacryloyloxy)ethyl]trimethylammonium chloride); (**c**) [3 (methacryloylamino)propyl]trimethylammonium chloride; (**d**) vinylbenzyl trimethylammonium chloride; and (**e**) diallyldimethylammonium chloride. Chain transfer agents: CTA1:4-cyano-4-[(dodecylsulfanylthiocarbonyl)sulfanyl]pentanoic acid), CTA2:4-cyano-4-(phenylcarbonothioylthio)pentanoic acid, CTA3:S-ethoxythiocarbonyl mercaptoacetic acid. Initiators: ACVA: 4,4-azobis(4-cyanovaleric acid), AAPH:2,2′-azobis(2-methylpropionamidine)dihydrochloride.

It is known that the antimicrobial activity of cationic polymers can be dependent on the type of monomer used and their hydrophobic content, charge density, molecular weight, and/or architecture [43,44,46,48]. The selected monomers have been reported as cationic blocks for copolymers to prepare surface coatings, hydrogels, and for gene delivery. Five cationic homopolymers, using commercially available ammonium-group-containing monomers, were hereby synthesized by RAFT polymerization, as described in Figure 1. The monomers were chosen as having either quaternary ammonium groups (diallyldimethyl ammonium chloride, [2-(methacryloyloxy)ethyl]trimethylammonium chloride, vinylbenzyl trimethylammonium chloride, 3-[(methacryloylamino)propyl]trimethylammonium chloride) or a tertiary amine (2-(dimethylamio)ethyl methacrylate, which will be protonated at physiological pH (average pKa of 7.5)) [66]. 

PDMAEMAs and their antibacterial actions have been studied in the literature [51,55]. It has been reported that lower-molecular-weight polymers can penetrate into Gram-positive bacteria more efficiently than their higher-molecular-weight counterparts, with cationic polyacrylates (5–10 kDa) optimal for antimicrobial activity against *S. aureus* [67]; however, there is little else known about the effect of their molecular weights on their activity or toxicity in biomedical applications. Here, we synthesized homopolymers using five different monomers, initially looking at polymers with a molecular weight of 20 kDa, before the synthesis of the chosen polymers with three different molecular weights (10, 20, and 40 kDa) to explore their antibacterial activity/mechanism and toxicity towards mammalian cells. GPC analysis of the polymers typically showed narrow, unimodal peaks with the polymerizations carried out in an aqueous environment, with the exception of 2-(dimethylamino)ethyl methacrylate, which was polymerized in ethanol (see Table 1). 

The polymerization of [2-(methacryloyloxy)ethyl]trimethylammonium chloride in water with 4-cyano-4-[(dodecyl sulfanyl thiocarbonyl)sulfanyl]pentanoic acid) (CTA1) was not achieved due to the limited solubility of CTA1 in aqueous environments. Water was the best solvent for the chain transfer agent 4-cyano-4-(phenylcarbonothioylthio) pentanoic acid (CTA2) with [2-(methacryloyloxy)ethyl]trimethylammonium chloride and gave the polymer in 5 h with 95% monomer conversion. GPC analysis showed a narrow, unimodal peak for the polymers (Mn calc 39.7 kDa, Mn GPC 37 kDa, Mw 39 kDa, PDI 1.03). It is worth noting that this is the first reported homopolymer synthesis of poly [2-(methacryloyloxy)ethyl]trimethylammonium chloride (PMEATCL) in the presence of the chain transfer agent 4-cyano-4-(phenylcarbonothioylthio)pentanoic acid and the water-soluble azo initiator 4,4-azobis (4-cyanovaleric acid). Similarly, the polymerization of vinyl benzyl trimethylammonium chloride was achieved with high conversion of the monomer to the polymer under similar conditions (Mn calc: 37.5 kDa, Mn GPC 23 kDa, Mw GPC 27 kDa, PDI 1.18). Meanwhile, 10 and 20 kDa polymers of PVMBT and PMETACL were synthesized by changing the [chain transfer agent]/[initiator] ratios. The polymerization of 3-[(methacryloylamino)propyl]trimethylammonium chloride was carried out under similar conditions; however, only 44% conversion was obtained after 24 h reaction (giving a polymer of approximately 20 kDa). GPC analysis of PVMBT and PMET3 showed polymers smaller than those calculated, perhaps explained by the structural differences in these homopolymers and the standard polymers used in GPC calibration (polyethylene glycols). 

The RAFT polymerization of diallyldimethylammonium chloride was challenging due to poor monomer reactivity; indeed, monomers such diallyldimethylammonium chloride are typically classified as “less activated” and require xanthate- or dithiocarbamate-based chain transfer agents [68]. Thus, the water-soluble xanthate-based chain transfer agent S-ethoxythiocarbonyl mercaptoacetic acid (CTA3) was synthesized [69] by reacting potassium ethyl xanthogenate with bromoacetic acid (91% yield) (see Figure S1), and the polymerization was carried out at 60 °C in the presence of the chain transfer agent and the water-soluble azo initiator 2,2′-azobis(2-methylpropionamidine)dihydrochloride. GPC analysis showed a narrow, unimodal peak for the polymer (Mn 18 kDa and Mw 25 kDa (PDI of 1.40) at 62% conversion (after 24 h). Figure 1 shows the details of the polymerization conditions and GPC analysis of the polymers (NMR spectra of the synthesized polymers are provided in the Supplementary Materials, Figures S2–S6).

### 2.2. Minimum Inhibitory Concentrations of the Polymers

The minimum inhibitory concentrations (MIC) of the polymers were determined against Gram-negative (*B. subtilis*, *E. coli*) and Gram-positive (*M. luteus*, *S. typhimurium*) bacteria by the resazurin-based microtiter viability assay. The blue dye, resazurin (7-hydroxy-3H-phenoxazin-3-one-10-oxide), can be irreversibly reduced to the pink, and highly red fluorescent, resorufin, by oxidoreductase within viable cells (resorufin can be further reduced to a colorless and non-fluorescent molecule, hydroresorufin). Briefly, serially diluted polymers were added to bacterial cultures to assess the concentration at which bacterial growth ceased. Color changes were observed visibly and spectrometrically. All assays were carried out in triplicate and the results of this are shown in Table 2. The MICs varied in the range of 16–64 µg/mL (Table 2). Expressing the data in terms of molarity (based on the average molecular weights of the polymers) displays their comparative strengths in an alternative manner and perhaps enables a fairer comparison between polymers with different molecular weights, and with conventional antibiotics, which have much lower molecular weights. As can be seen from Table 2, PDADMAC showed the best inhibition against all bacteria screened. 

The toxicity of the homopolymers was analyzed on HeLa cells via an MTT assay 1.25–10 µM (25–200 µg/mL—see Figure S7). This showed that PMETACL and PVMBT were the least toxic cytotoxic polymers (to HeLa cells), and since neither of these homopolymers has been investigated previously, with respect to antimicrobial activity, they were selected alongside PDADMAC for further investigation.

### 2.3. Investigation of the Molecular Weight and Antimicrobial Activity

The antimicrobial activity of cationic polymers is influenced by several factors, including the molecular weight, the hydrophobicity/hydrophilicity balance, the positions of cationic units, and the polymer architecture [13,70]. Literature [71] suggests that the larger the polymer (Mw > 100 kDa), the greater the biocidal effects, until supposed permeability limitations come into play and activity falls. However, such studies have typically used polydisperse polymers. Here, we targeted monodisperse polymers with molecular weights between 10 and 40 kDa, rationalizing that smaller polymers would be more likely to penetrate into bacteria while the largest polymers might be expected to interact with the outer membranes of Gram–negative bacteria, although the cumulative effect of additional monomer units could contribute to the antibacterial properties of the polymers. Small polymers have advantages over small molecules as, due to their mechanisms of action, they are unlikely to be subjected to classic resistance mechanisms. In this context, three different molecular weights of PDADMAC, PMETACL, and PVBMT were synthesized via RAFT polymerization (Table 3) (for GPC analysis of polymers see Figure S8).

The minimum inhibitory concentration of the low (10 kDa), medium (20 kDa), and high (40 kDa) molecular weights of a particular polymer at three different concentrations were evaluated (see Table 4) and showed that the higher-molecular-weight polymers were more effective in inhibiting bacterial growth, an effect confirmed by an agar diffusion assay against all four tested bacterial species (see Figure S9, Table S1).

Among the tested polymers, PDADMAC-40 showed the best inhibition of all bacteria strains, with an MIC in the range of 0.43–0.86 µM (16–32 µg/mL). However, it is worth mentioning that increased polymer chain length was also found to lead to enhanced cytotoxicity, with PDADMAC-40 being highly cytotoxic to HeLa cells even at the lowest concentration (1.25 µM) tested. Importantly, PDADMAC-20, PMETACL-40, and PVMBT-40 showed similar MIC values (0.9 µM), but were much less cytotoxic.

### 2.4. Antimicrobial Mechanisms of the Polymers

**Figure 2:**
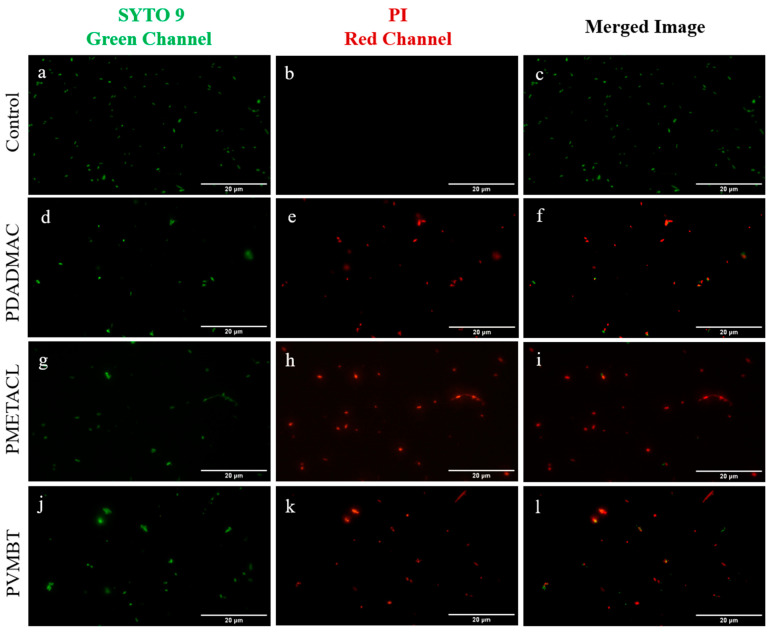
Fluorescent microscopy images of *E. coli* stained using a live/dead assay (live are shown in green (SYTO 9) and dead are shown in red (PI)). Control bacteria (**a**–**c**) and bacteria following treatment with the three lead polymers (PDADMAC (**d**–**f**); PMETACL (**g**–**i**) and PVMBT (**j**–**l**)). Bacteria were imaged on a Zeiss Axiovert 200M, with a 40× objective, in the FITC channel (λex 488 nm) (**a**,**d**,**g**,**i**) and the Texas red channel (λex 561 nm) (**b**,**e**,**f**,**j**) with the merged images shown (**c**,**f**,**h**,**k**). Red fluorescence indicates loss of membrane integrity and signifies the bactericidal action of the compound (scale bar: 20 µm).

A live/dead assay was used for the assessment of bacterial membrane integrity after treatment with the polymers, with double staining using SYTO 9 and propidium iodide (PI). Figure 2 shows images of bacteria after a 4-h incubation period with each of the three polymers (PDADMAC-20, PMEATCL-40, and PVMBT-40) at 2× MIC (Figure 2). The loss of membrane integrity (structural disturbance of the membrane) signified the bactericidal action of the compounds, the generally accepted mechanism for ammonium-group-bearing polymers [13].

**Figure 3:**
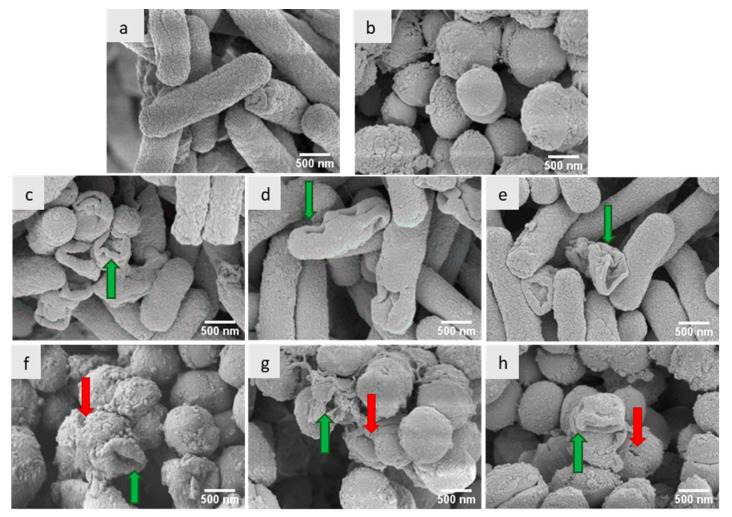
SEM images of (**a**) untreated *E. coli*; (**c**) PDADMAC-20-treated *E. coli*; (**d**) PVMBT-40-treated *E. coli*; and (**e**) PMETACL-40-treated *E. coli*. (**b**) Untreated *M. luteus*; (**f**) PDADMAC-20-treated *M. luteus*; (**g**) PVMBT-40-treated *M. luteus*; and (**h**) PMETACL-40-treated *M. luteus*. Green arrows indicate bacteria with impaired membranes. Red arrows indicate the pores on the bacterial surface of *M. luteus*. Scale bars: 500 nm.

In order to understand the mechanism of action, *E. coli* and *M. luteus* were treated with the most active polymers (PDADMAC-20, PVMBT-40, and PMETACL-40) and analyzed by SEM to observe the bacterial morphology. As shown in Figure 3, the distortion of the bacteria suggested that the polymers were disrupting/distorting the bacterial envelopes of both Gram-positive and Gram-negative bacteria, with changes in membrane roughness, shrinking, and involutions. The *E. coli* “outer envelope” showed wrinkles and deep hollows, while a misshaped and raptured membrane of *M. luteus* was observed. Moreover, pores were found on the surface of *M. luteus*, suggesting the permeabilization of the plasma membrane and leakage of cellular content. These phenomena suggest that these polymers kill bacteria by destroying their cell membranes.

### 2.5. Membrane Depolarization Assays

**Figure 4:**
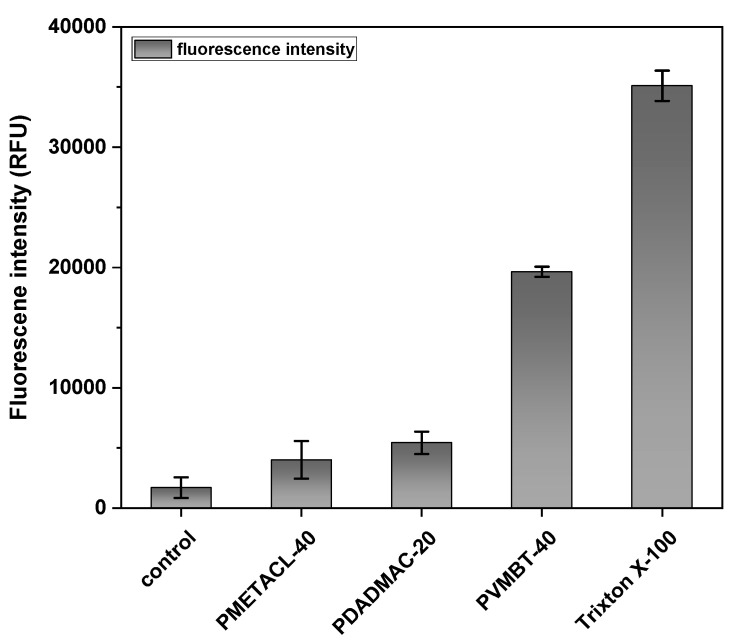
Permeabilization of the outer membrane of *E. coli* was measured using a 1-N-phenyl-naphthylamine (NPN) assay in the presence of polymers PVMBT-40, PDADMAC-20, and PMETACL-40 after 20 min. Bacteria were incubated with NPN for 30 min to stabilize the fluorescence (data are not shown) and polymers added at 4× MIC (3.6 µM). Bacteria without polymer treatment were used as a negative control, while Triton X-100 (1% *v*/*v*)-treated bacteria served as the positive control (n = 3).

**Figure 5:**
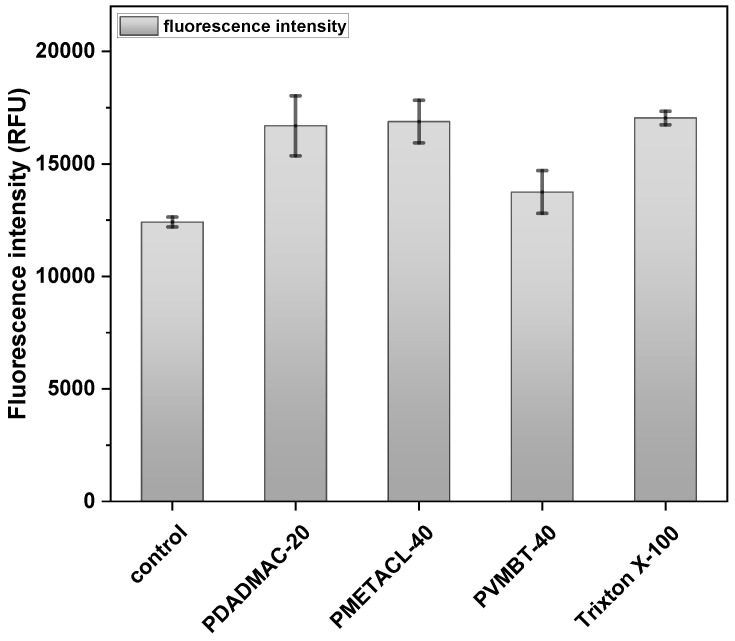
Permeabilization of the membrane of *B. subtilis* by polymers PVMBT-40, PDADMAC-20, and PMEATCL-40 (at 4× MIC) and the increase in fluorescence of calcein monitored over 30 min. Calcein-AM-loaded bacteria without polymer treatment were used as a negative control, while Triton X-100 (1% *v*/*v*)-treated bacteria served as the positive control (n = 3).

Perturbation of the bacterial outer membrane (Gram-negative bacteria) was assessed using the fluorescent probe (1-N-phenyl-naphthylamine) (NPN) that enters and binds damaged membranes. Supported by the SEM images, the assay results suggested that the outer membrane of *E. coli* was depolarized by the polymers, with PVMBT-40 giving a 10-fold increase in fluorescence (Figure 4). Presumably, the ammonium groups accumulate on the membranes via electrostatic interactions, and the hydrophobic aryl groups interdigitate with the lipophobic membrane, promoting the interlacing of the polymer, leading to enhanced permeability. Gram-positive bacteria have a thick layer of crosslinked peptidoglycan decorated with negatively charged teichoic acid, surrounding their cytoplasmic membranes. Cationic polymers can accumulate onto this via electrostatic interactions and then penetrate deep into the peptidoglycan layer by virtue of nano-sized pores or defects. These accumulated cationic polymers can then disturb the integrity of the cytoplasmic membrane, leading to bacterial death. This effect of the polymers on Gram-positive bacteria (*B. subtilis*) was measured by the polymer-induced leakage of the fluorescent dye calcein. Analysis (Figure 5) showed that the polymers accessed the cytoplasmic membrane, even in the presence of the thick cell wall. PDADMAC-20 and PMETACL-40 showed a similar fluorescence increase to Triton X-100 (1% *v*/*v*), with PDADMAC-20 acting faster than PMEATCL-40, presumably due to its lower molecular weight. The more hydrophilic polymer chains of PMEATCL-40 penetrated into the peptidoglycan layer more efficiently than PVMBT-40.

### 2.6. Cytotoxicity and Hemolytic Activity of Polymers

**Figure 6:**
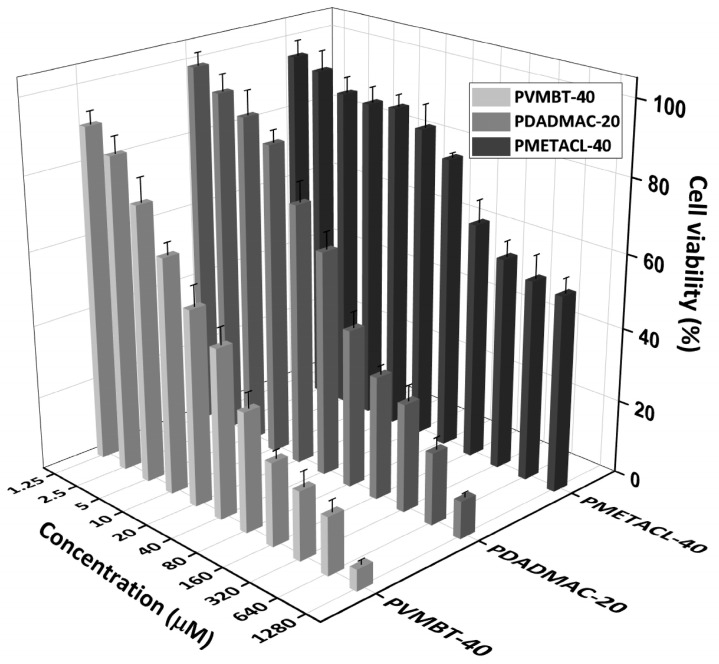
MTT cytotoxicity assay of the polymers PDADMAC-20, PMETACL-40, and PVMBT-40 at 1.25–1280 µM on HeLa cells for 24 h. Results are presented as relative cell viability compared to that of the untreated negative control (100% cell viability). The error bars are standard deviation of the mean (n = 3).

As shown above, the higher-molecular-weight polymers showed better antimicrobial activity. An MTT cytotoxicity assay showed that the homopolymers (see Figure 6) displayed greater toxicity with increasing concentration and the higher-molecular-weight polymers were more toxic than the corresponding lower-molecular-weight analogues (Figure S10) [60]. This is due to the multiple positively charged segments, interacting with components of the negatively charged membrane (in both extracellular and intracellular compartments), ultimately initiating apoptosis [60,63]. Much of this will be entropic- and polymer-flexibility driven—once bound, the adjacent cations will be in proximity to bind additional elements of the cell membrane. Figure 6 shows HeLa cell viability after incubation with polymers for 24 h, with the three homopolymers of PMETACL being the least toxic (Figure S10), even at 1280 µM (far beyond the antibacterial MIC of the polymer (16–32 µg/mL); over half the cells were viable).

**Figure 7:**
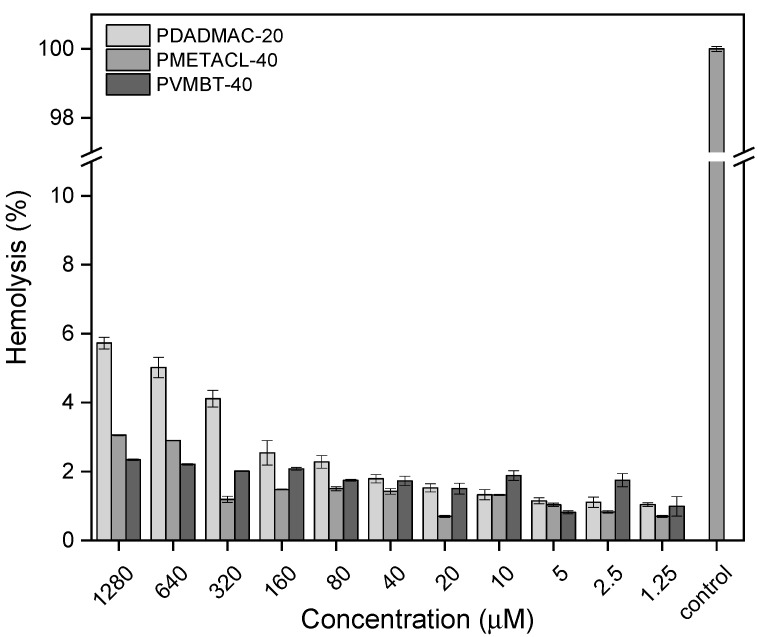
The hemolytic activity (sheep erythrocytes) of the polymers PDADMAC-20, PMETACL-40, and PVMBT-40. A hemolysis test was used to assess the biocompatibility of polymers with varying concentrations of the polymers. The amount of leaked hemoglobin (% hemolysis) was measured relative to a positive control (Triton X-100 (1% *v*/*v*) defined as giving 100% lysis of red blood cells and a negative control (PBS-treated red blood cells) giving no lysis.

The hemolytic activity was investigated as a biocompatibility indicator (PDADMAC-20, PMETACL-40, and PVMBT-40 (as seen in Figure 7)); all showed limited hemolysis, even at the highest concentrations used (1280 µM).

A practical approach to assessing selectivity towards bacterial vs. mammalian cells is to measure the HC_50_/MIC ratio, where HC_50_ is the polymer concentration required to lyse 50% of red blood cells [51], with many of the polymers described herein selective against bacteria over mammalian cells across the concentration range evaluated (Table 5).

## 3. Materials and Methods

All monomers and common chemical reagents, 2,2-azobis(2-methylpropionamidine)dihydrochloride (AAPH, 97%), potassium ethyl xanthogenate (96%), bromoacetic acid (97%), 4,4′-azobis(4-cyanopentanoic acid) (ACVA), 4-cyano-4-[(dodecylsulfanylthiocarbonyl)sulfanyl]pentanoic acid (CTA1), 4-cyano-4-(phenylcarbonothioylthio)pentanoic acid (CTA2), NaOH, anhydrous acetonitrile, ethanol, acetone, hexane, and deuterated solvents (D_2_O, CDCl_3_) were purchased from Sigma Aldrich (St. Louis, MO, USA), Fluorochem (Glossop, UK), Fisher Scientific (Hampton, NH, USA), or Alfa Easer (Haverhill, MA, USA) and used as received, unless otherwise noted. Except for diallyldimethylammonium chloride (65% wt. in H_2_O), all monomers were passed through a basic alumina column to remove inhibitors before polymerization reactions. The chain transfer agent, S-ethoxythiocarbonyl mercaptoacetic acid (CTA3), was synthesized according to the literature [69]. Deionized (DI) water was from a Milli-Q system. Dulbecco’s Modified Eagle Medium (DMEM), Opti-MEM (OMEM), 0.25% trypsin−EDTA, fetal bovine serum (FBS), and streptomycin (5000 μg/mL)/penicillin (5000 U/mL) were obtained from ThermoFisher Scientific, Horsham, UK, while 3-[4,5-dimethylthiazol-2-yl]-2,5 diphenyltetrazolium bromide was purchased from Alfa Easer. Phosphate buffer solution (PBS) was prepared from PBS tablets (Oxoid Ltd., ThermoFisher Scientific, Horsham, UK). Defibrinated sheep blood (SB054-100 mL) was provided by TCS Biosciences. The LIVE/DEAD™ BacLight™ Bacterial Viability Kit for microscopy was purchased from Sigma-Aldrich. Calcein-AM was purchased from Sigma-Aldrich. N-Phenyl-1-naphthylamine (98%) was purchased from Thermo Scientific. *Escherichia coli* (DH5α), *Salmonella typhimurium* SLI344, *Micrococcus luteus* (ATCC 4698), and *Bacillus subtilis* (ATCC 6051) were used as Gram-positive and Gram-negative test strains. Microbial culture broths and agar medium (Invitrogen, Inchinnan, UK; Fisher Scientific, UK; and Merck, Inchinnan, UK), buffers, and water were sterilized by autoclaving before use. Polymer solutions were filter-sterilized (0.22 µ, Millipore Inc., Burlington, MA, USA) before use. 

### 3.1. Methods

#### 3.1.1. General Synthesis of Homopolymers via Reversible Addition Fragmentation Chain

##### Transfer Polymerization

Monomers were subjected to free radical polymerization to determine the reaction conditions and understand the compatibility of monomers, initiators, and solvent. Subsequently, all monomers were subjected to RAFT polymerization with a suitable chain transfer agent. During the polymerization reactions, aliquots were taken to follow monomer conversion (by ^1^H NMR spectrometry in D_2_O or CDCl_3_). The experimental molecular weights of the polymers were calculated based on the integration of the disappearing vinyl protons of the responsible monomer and the appearance of the methylene peak of the formed polymer via ^1^H NMR. Theoretical molecular weights were calculated according to the literature [50] based on the formula below, where [M]_0_, and [CTA]_0_ are the initial concentrations of the monomer and chain transfer agent; *p* is the monomer conversion; and M_wM_ and M_wCTA_ are the molar masses of the monomer and chain transfer agent, respectively.

$$M_{n,th}=\left(\frac{{\left[M\right]}_{0}\cdot p\cdot {Mw}_{M}}{{\left[CTA\right]}_{0}}\right){+Mw}_{CTA}$$

The monomer DADMAC was polymerized using the initiator 2,2′-azobis (2-methylpropionamidine) dihydrochloride (AAPH) and the xanthate-type chain transfer agent S-ethoxythiocarbonyl mercaptoacetic acid (CTA3) at 60 °C. The other monomers were polymerized using the initiator 4,4-azobis (4-cyanovaleric acid) (ACVA) and either 4-cyano-4-(phenylcarbonothioylthio) pentanoic acid (CTA2) or 4-cyano-4-[(dodecyl sulfanyl thiocarbonyl)sulfanyl]pentanoic acid) (CTA1) at 70 °C as the chain transfer agents. In order to synthesize the desired different molecular weights of the polymers, the chain transfer and initiator concentrations in the reaction were tuned while the monomer concentrations were kept constant (Figure 1).

#### 3.1.2. P1-RAFT Polymerization of Poly(2-(dimethylamino)ethyl methacrylate)

First, 2-(dimethylamino)ethyl methacrylate (5 mL), radical initiator 4,4′-azobis(4-cyanopentanoic acid) (ACVA), (0.0084 g), chain transfer agent CTA1-(4-cyano-4-[(dodecylsulfanyl thiocarbonyl)sulfanyl] pentanoic acid) (0.0726 g), and 10 mL ethanol were mixed in a septum sealable glass tube (50 mL). The solution was degassed by argon bubbling for 30 min. The polymerization was carried out at 70 °C for 24 h. The polymer was purified by removal of the ethanol, redissolution in THF, and precipitation using n-hexane. The polymer was dried under vacuum at 40 °C for 2 days to give a yellow-colored polymer (78% yield).

#### 3.1.3. P2-RAFT Polymerization of Poly([2-(methacryloyloxy)ethyl]trimethylammonium chloride)

First, [2-(methacryloyloxy)ethyl]trimethylammonium chloride) (6 g, 75% wt in H_2_O), initiator 4,4-azobis (4-cyanovaleric acid (8 mg), raft agent CTA2 (40 mg), and water (17.9 mL) were mixed in a 50 mL Schlenk flask and the reaction solution was purged with nitrogen for 30 min. The polymerization reaction was carried out at 70 °C for 24 h. Aliquots were taken at different time intervals to check monomer to polymer conversion via ^1^H NMR analysis. The resulting polymer was purified by precipitation using acetone, redissolved in methanol, and again precipitated using acetone and dried under vacuum at 40 °C to give a salmon-colored polymer (94% yield). This polymer was synthesized with three different molecular weights by changing the chain transfer agent to initiator ratio. 

#### 3.1.4. P3-RAFT Polymerization of Poly[3-(methacryloylamino)propyl]trimethylammonium chloride

First, [3-(methacryloylamino)propyl]trimethylammonium chloride (13 g 50% wt in H_2_O), initiator 4,4-azobis(4-cyanovaleric acid (8 mg), CTA2 (40 mg), and water (17.5 mL) were mixed in a 100 mL flask and the reaction solution was purged with nitrogen for a 30 min. It was then placed into a preheated oil bath at 70 °C for 24 h. The resulting polymer was purified by precipitation with excess acetone, redissolved in methanol, re-precipitated with acetone, and dried under vacuum at 40 °C (51% yield).

#### 3.1.5. P4-RAFT Polymerization of Poly(vinylbenzyl trimethylammonium chloride)

The monomer (4 g), 4,4-azobis(4-cyanovaleric acid) (ACVA) as an initiator (7.84 mg), CTA2 (28 mg), and water (25 mL) were mixed in a 50 mL Schlenk flask and purged with nitrogen for 30 min. The polymerization reaction was carried out at 70 °C for 24 h. The resulting polymer was purified by precipitation using THF and dried under vacuum at 40 °C (89% yield). This polymer was synthesized with three different molecular weights by changing the changing the chain transfer agent to initiator ratio.

#### 3.1.6. P5-RAFT Polymerization of Poly(diallydimethyl ammonium chloride)

The polymerization of diallyldimethyl ammonium chloride was carried out by the protocol of Demarteau [72]. Diallyldimethyl ammonium chloride (10 mL, 65% wt in H_2_O), initiator 2,2′-azobis (2-methylpropionamidine)dihydrochloride (AAPH) (0.019 g), CTA3 (0.041 g), and water (8 mL) were mixed in a 50 mL Schlenk tube. Then, the reaction mixture was degassed by bubbling with argon for 30 min and placed in preheated oil bath at 60 °C for 24 h. The resulting polymer was precipitated in a mixture of acetone/ethanol (1:1) three times and dried under vacuum at 40 °C (70% yield). This polymer was synthesized with three different molecular weights by changing the chain transfer agent to initiator ratio.

The synthesized polymers were numbered as follows: PDMAEMA (P1), PMETACL (P2), PMET3 (P3), PVBMT (P4), and PDADMAC (P5).

#### 3.1.7. Synthesis of Chain Transfer Agent S-Ethoxythiocarbonyl Mercaptoacetic Acid (CTA3)

Water-soluble xanthate-type chain transfer agent CTA3 was synthesized according to the literature [69]. Sodium hydroxide (1.25 g, 62.4 mmol) was dissolved in chilled water (50 mL) and then bromoacetic acid (1 eq, 4.37 g, 62.4 mmol) was added until a clear solution was obtained. Potassium ethyl xanthogenate (1 eq, 5 g, 62.4 mmol) was then added to the mixture over 30 min. The solution was stirred at room temperature for 24 h and then acidified with 4 M HCl. The resulting mixture was extracted with chloroform (3 × 50 mL). The organic extracts were dried over anhydrous magnesium sulfate, filtered, and concentrated under vacuum. The solid was washed with hexane and dried under vacuum to give white crystals. ^1^H NMR (500 MHz, D_2_O) δ 4.63 (q, *J* = 7.1 Hz, 2H, CH_2_), 3.93 (s, 2H, CH_2_), 1.34 (t, *J* = 7.1 Hz, 3H, CH_3_). ^13^C NMR (126 MHz, D_2_O) δ 213.95, 172.80, 71.72, 37.32, 12.79) (Figure S1b). LC-MS (ESI) for C_5_H_8_O_3_S_2_: [M–H]^+^ calcd.: 179.1; found: 179.1. Data were consistent with the literature [69].

### 3.2. Characterization of Homopolymers

#### 3.2.1. Nuclear Magnetic Resonance Spectroscopy

All monomers, polymers, and RAFT agents were analyzed on a Bruker AVA500 spectrometer in CDCl_3_ or D_2_O at either 500 MHz (H^1^ NMR) or 125 MHz (C^13^).

#### 3.2.2. Molecular Weight Determination of Polymers by GPC analysis

Aqueous-based GPC analysis of the polymers was carried out on an Agilent Technologies 1100 system, 8 μm Agilent PL Aquagel-OH 30, and 8 μm Agilent PL Aquagel-OH 40 columns with an RI detector. The eluent was 0.50 M acetic acid, 0.30 M NaH_2_PO_4_, at pH 2.5, with a flow rate of 1.0 mL min^−1^ at 25 °C. Calibration was achieved using InfinityLab EasiVial poly(ethylene oxide) standards with Mn values ranging from 1.1 to 905 kDa. GPC analysis of poly (2-(dimethylamino)ethyl methacrylate) (PDMAEMA) was carried out on an Agilent 1260 Infinity system on two PL-GEL mixed-c columns (5 μm) with both UV and RI detectors. The eluent used was THF at a flow rate of 1.0 mL min^−1^ at 35 °C. Molecular weights were obtained relative to poly(methyl methacrylate) standards.

### 3.3. Antimicrobial Activity of the Polymers

#### 3.3.1. Primary Screening: Determination of Minimum Inhibitory Concentration

Antibacterial activity was assessed using a resazurin colorimetric assay [73]. Five µL of a cryopreserved glycerol stock of the bacterial culture (*Escherichia coli*, *Salmonella typhimurium*, *Micrococcus luteus*, and *Bacillus subtilis*) was streaked onto Luria–Bertani, nutrient agar, tryptic soy agar, and nutrient agar plates, respectively. A single bacterial colony was transferred to 5 mL of the respective broth medium and incubated at 37 °C and grown until the mid-log phase. The culture was diluted and the concentration adjusted to 0.5 McFarland standard with sterile Mueller–Hinton broth (2 × 10^7^ CFU/mL). The resazurin solution was prepared in a brown glass vial by dissolving 34 mg of resazurin in sterile distilled water (5 mL) with vortexing for 1 h to ensure homogeneity. A single 96-well microtiter plate (Corning, black 96-well flat-bottomed plates) was dedicated to each bacterial species to prevent contamination. The design of the assay was prepared with two rows and two columns from each end filled with sterile water to avoid edge effects. The assay was adapted from previously reported protocols [73]. Two columns of broth sterility controls, 1 column of a growth control, 2 columns of polymer sterility controls (P1-5 with one polymer in each well), 1 column of a positive antibiotic control, and, lastly, 2 columns of polymer test samples (P1-5 with one polymer in each well) were used. All wells were filled with 100 µL of Mueller–Hinton broth. The broth sterility and growth control columns contained 100 µL of sterile water; the polymer sterility control and test wells contained 100 µL of polymers (final concentration 128 µg/mL) in the designated wells. Antibiotic wells, all at 64 µg/mL, contained chloramphenicol (for S. *typhimurium*), gentamicin (for *E. coli* and *M. luteus*), and clindamycin (for *B. subtilis*). Five μL of the diluted bacterial suspension (2 × 10^7^ CFU/mL) was added into all wells (except the broth sterility and polymer sterility control columns) and mixed thoroughly. After overnight incubation at 37 °C, resazurin solution (5 μL, 6.75 mg/mL) was added to all wells and incubated at 37 °C for another 4 h. Changes in color from blue (resazurin, no bacterial growth) to pink (resorufin, bacterial growth) were recorded at 595 nm on a microplate reader (BioTek Synergy HT). The lowest concentration that did not show a color change was considered as the minimum inhibitory concentration (MIC). Each assay was performed in triplicate.

#### 3.3.2. Effect of Molecular Weight on Antimicrobial Activity of Polymers

In order to determine the effect of the molecular weight on the antibacterial activity, three differently sized polymers were synthesized for PDADMAC, PMETACL, and PVBMT.

*Zone diffusion assays and growth kinetics***:** Agar plates were inoculated by spreading 100 µL bacteria (1 × 10^6^ CFU/mL), 1-cm-diameter wells were punched into the agar, and 100 µL of polymer solution (at 2× MIC) was added into the wells and the zones of inhibition were measured after 8 h.

### 3.4. Live/Dead Assay

Bacterial cultures (5 mL) grown to the late log phase were harvested by centrifugation. The cell pellet was resuspended in 5 mL 0.85% NaCl, aliquoted (each aliquot was 1 mL), and incubated with 4× MIC concentrations of the selected polymers for 4 h at 37 °C. The live cell control consisted of an aliquot incubated with only 0.85% NaCl. After incubation, all samples were washed with 0.85% NaCl twice. A Live/Dead BacLight Bacterial Viability Kit (Invitrogen) was used to check cell viability. The assay was performed according to the kit instructions. Component A (200 µL of a 3.34 mM solution in DMSO) was mixed in equal proportions with Component B (200 µL of a 20 mM solution in DMSO) and 3 μL of this combined dye mixture was added to 1 mL of all bacterial samples before incubation for 30 min in the dark. Samples were analyzed using an AxioVert 200M inverted fluorescent microscope to analyze for green (λex 488 nm, λem 520 nm) and red (λex 561 nm, λem 646 nm) fluorescence.

### 3.5. Bacterial Membrane Integrity Assays

*Outer membrane permeabilization assay:* The method for the determination of the outer membrane depolarization assay was adapted from the literature [74]. First, 1-N-phenyl-1-naphthylamine was dissolved in acetone (3 mM). Gram-negative bacteria *E. coli* were grown to 1.0 OD. Cells were harvested by centrifugation, washed, and resuspended to 0.1 OD in 5 mM HEPES with 5 mM glucose at pH 7.2. Then, 10 µL of NPN stock was added to each 1 mL of bacteria to give a concentration of NPN of 30 µM. Next, 200 µL of bacterial suspension was added to each well of a 96-well plate as five replicates at 37 °C, followed by analysis on a fluorescent microplate spectrometer (BioTek Synergy HT) over 30 min (every 1 min) (λex 350 nm λem 429 nm). Then, 10 µL of polymer solution (4× MIC) was added to each well. Moreover, 1% *v*/*v* Triton X-100 was used as a positive control, while a bacterial suspension without polymer was used as a negative control. The increase in the fluorescence of NPN was monitored at 5-min intervals and the fluorescence values after 30 min plotted. All experiments were repeated three times independently.

*Cytoplasmic depolarization assay:* The method for the determination of the inner membrane depolarization assay was adapted from the literature [75]. A stock solution of calcein-AM was prepared in DMSO (1 mM) and stored in the dark at −20 °C. *B. subtilis* was cultured in media overnight and centrifuged (3000× *g*—5 min) to give a pellet. The pellet was washed with PBS and resuspended to a 0D600 of 1.0 with PBS containing 10% (*v*/*v*) broth. The bacteria were incubated with 3 µM calcein-AM for 1 h at 37 °C. The dye-loaded cells were collected by centrifugation (3000× *g*—5 min) and suspended to 0.1 OD in PBS. Then, 200 µL of bacterial suspension was added to sterile black-wall 96-well plates (three replicates) and monitored for 30 min (λex 622 nm, λem 670 nm) on a fluorescence plate reader. Then, 10 µL of polymer solution (4× MIC) was added to each well. Bacteria without polymers were used as a negative control, while 1% *v*/*v* Triton X-100 was used as a positive control. All experiments were repeated three times independently.

### 3.6. Scanning Electron Microscopy

Aliquots (10 mL of OD 0.5 of Gram-positive (*M. luteus*) and Gram-negative (*E. coli*)) of bacteria were incubated with 0.85% NaCl (control) and the polymers PDAMAC-mid (20 kDa), PVMBT-high (40 kDa), and PMETACL-high (40 kDa) (all 2× MIC) for two hours. After pelleting, the bacteria were fixed in a solution of 3% glutaraldehyde in 0.1 M sodium cacodylate buffer (pH 7.3) for 2 h and washed (3 × 10 min changes of 0.1 M sodium cacodylate buffer). Samples were then post-fixed in 1% osmium tetroxide (in 0.1 M sodium cacodylate buffer) for 45 min, before further 3 × 10 min washes were performed in 0.1 M sodium cacodylate buffer. Dehydration in graded concentrations of acetone (50%, 70%, 90%, and 3 × 100%—each for 10 min) was followed by critical point drying using liquid carbon dioxide. After mounting on aluminum stubs with carbon tabs attached, the specimens were sputter-coated with 20 nm thickness of gold–palladium and viewed using a Hitachi S-4700 scanning electron microscope.

### 3.7. MTT Assay

In vitro cytotoxicity tests were carried out using an MTT assay on HeLa cells, based on a previously published protocol, with some modifications [76]. Cells were grown in complete medium (Dulbecco’s Modified Eagle Medium, DMEM, Gibco) supplemented with 2 mM L-glutamine, 10% fetal bovine serum, and 1% antibiotics (penicillin 100 U/mL and streptomycin 100 μg/mL) until sub-confluent at 37 °C and 5% CO_2_ in a cell incubator (HERAcell ^®^150, Kendro, Hereaeus Group, Hagen, Germany). HeLa cells were used at passage numbers below 15 and tested for mycoplasma contamination regularly. For cytotoxicity tests, cells were seeded in a 96-well plate at the density of 1 × 10^4^ cells per well and cultured in a 100 μL of growth medium. After 24 h, the culture medium was replaced with polymer solutions (50 µL) prepared in growth media at increasing concentrations. The cells were further incubated for 24 h under the same conditions. After the incubation of cells with polymers containing media, all the solutions were carefully removed from the wells and 100 µL MTT solution (5 mg of MTT dissolved in 1 mL PBS and 9 mL serum-free DMEM without phenol-red) was added to each well in a light-protected environment. After 4 h, unreacted dye was removed by aspiration. Cells were carefully washed with PBS twice. Then, 100 μL of solubilizing solution (a mixture of 2-propanol and dimethyl sulfoxide in a 1:1 volume ratio) was added to each well to dissolve the formed formazan crystals. The solution was shaken for at least 30 min to ensure the dissolution of all crystals before measuring the absorbance at 570 nm on a BioTek Synergy H1 plate reader. Cells treated with complete medium only were taken as a positive control (100% viability) and cells treated with DMSO (100%) were taken as a negative control. All tests were repeated three times. The relative cell viability (%) with respect to the control was determined using the formula below:

$$Cell\;viability\left(\%\right)=\left(\frac{{OD}_{sample}-{OD}_{negative\;control}}{{OD}_{positive\;control}-{OD}_{negative\;control}}\right)\times 100$$

where OD is the optical density at 570 nm.

The test was applied in the range of 1.25 µM to 1280 µM for the polymers (PDADMACs, PMEATCLs, and PVMBTs).

### 3.8. Hemolysis Assays

Assays were carried out following previously published protocols [77]. Sheep red blood cells (RBCs) were diluted 1:20 in PBS (pH 7.4), pelleted by centrifugation, and washed three times in PBS (20 mL, 1000 g, 10 min). The RBCs were then resuspended to 5% (*v*/*v*) in PBS. Different concentrations of PDAMAC-mid (20 kDa), PVMBT-high (40 kDa), and PMETACL-high (40 kDa) (150 µL, in the range of 1.25–1280 µM) were prepared, followed by the addition of the 5% RBC suspension (150 µL). PBS buffer was used as a negative control, and Triton X-100 (1% *v*/*v* in PBS) was used as a positive hemolysis control. Tubes were incubated at 37 °C for 1 h with 300 rpm shaking in an incubator. Samples were then centrifuged (2000× *g*, 4 min), and 100 μL aliquots of supernatants were transferred into a 96-well microplate, where absorbance values were read at 405 nm using a plate reader. The hemolysis percentage was calculated using the absorbance values and the formula below:

$$Hemolysis\left(\%\right)=\left(\frac{A_{polymer}-A_{negative}}{A_{positive}-A_{negative}}\right)\times 100$$

where A_polymer_ is the absorbance of the polymer-treated cells’ supernatant, A_negative_ is the absorbance of the negative control (1% *v*/*v* Triton X-100-treated cells’ supernatant), and A_positive_ is the absorbance of the positive control (PBS-treated cells’ supernatant). All experiments were performed as independent triplicates, each consisting of triplicates.

## 4. Conclusions

In this study, five quaternary homopolymers were screened on Gram-negative and Gram-positive bacteria to detect the most active antimicrobial polymer. Selected polymers (PDADMACs, PVMBTs, and PMETACLs) were synthesized with varying molecular weights (~10, ~20, and ~40 kDa) and with the higher-molecular-weight polymers showing (on a mole/mole bases) higher activity (lower MIC values). PVMBT (40 kDa) and PMETACL (40 kDa) and PDADMACs (20 kDa) showed good growth inhibition against both Gram-positive (*B. subtilis*, *M. luteus*) and Gram-negative (*S. typhimurium*, *E. coli*) bacteria, with MIC values as low as 0.9 µM. The antimicrobial action mechanisms of the polymers were determined using dye permeabilization assays and visualization by SEM, which showed the formation of “holes” on the bacteria and a highly distorted cell envelope. Cytotoxic evaluations of the polymers were carried out using MTT and hemolysis assays, and the best polymers showed great selectivity towards bacteria over eukaryotic cells. Overall, the polymer PMETACL, in its higher-molecular-weight form, showed strong inhibition of bacterial growth (MIC = 0.9–1.8 µM (16–32 µg/mL)), with a bactericidal mode of action against both Gram-negative and Gram-positive bacteria, and it also showed low mammalian cell toxicity, making it a potential candidate for biomedical applications.

## Figures and Tables

**Table 1:** Cationic homopolymers synthesized via RAFT polymerization. Monomer to initiator ratios, chain transfer agent to initiator ratios, calculated molecular weights (obtained from monomer conversions as analyzed by ^1^H NMR and molecular weight obtained by GPC), and PDI values.

Code	Polymer	CTA	[M]/[I]	[CTA]/[I]	Mn Calc.	Mn-GPC	Mw-GPC	PDI
P1	PDMAEMA	1	167	6	23.0 kDa	22 kDa	28 kDa	1.29
P2	PMETACL	2	100	5	17.1 kDa	17 kDa	20 kDa	1.19
P3	PMET3	2	215	5	23.1 kDa	14 kDa	16 kDa	1.18
P4	PVMBT	2	190	7	20.8 kDa	14 kDa	16 kDa	1.11
P5	PDADMAC	3	175	3.3	17.5 kDa	18 kDa	24 kDa	1.36

**Table 2:** Minimum inhibitory concentrations of the synthesized homopolymers. Concentrations in µM are based on the average molecular weight of the polymer.

Polymer	Code	MICs for Different Target Microorganisms							
		*B. subtilis*		*E. coli*		*M. luteus*		*S. typhimurium*	
		µg/mL	µM	µg/mL	µM	µg/mL	µM	µg/ mL	µM
PDMAEMA	P1	32	1.6	32	1.6	32	1.6	32	1.6
PMETACL	P2	64	1.9	64	1.9	64	1.9	64	1.9
PMET3	P3	32	1.4	32	1.5	16	0.7	64	2.8
PVBMT	P4	32	0.9	64	1.7	64	1.7	64	1.7
PDADMAC	P5	16	0.9	16	0.9	16	0.9	32	1.8
Standard Antibiotics		Clindamycin		Gentamicin		Gentamicin		Chloramphenicol	
		4	9.4	0.5	1.1	0.5	1.1	1	3.1

**Table 3:** Properties of PDADMAC, PMETACL, and PVMBT synthesized with different molecular weights (approx. 10 kDa, 20 kDa, and 40 kDa) via RAFT polymerization using CTA2 for the PMETACLs and PVMBTs and CTA3 for the PDADMACs in aqueous media.

Polymer	Mn Calc.	Mn-GPC	Mw-GPC	PDI
PMETACI-10	10.4 kDa	11 kDa	14 kDa	1.24
PMETACI-20	17.1 kDa	17 kDa	20 kDa	1.19
PMETACI-40	39.7 kDa	39 kDa	40 kDa	1.03
PDADMAC-10	11.9 kDa	11 kDa	15 kDa	1.36
PDADMAC-20	17.5 kDa	18 kDa	24 kDa	1.36
PDADMAC-40	36.8 kDa	35 kDa	48 kDa	1.37
PVMBT-10	13.7 kDa	6 kDa	7 kDa	1.20
PVMBT-20	20.8 kDa	14 kDa	16 kDa	1.11
PVMBT-40	37.4 kDa	23 kDa	27 kDa	1.18

**Table 4:** Effect of molecular weight on the polymers’ MICs as determined by a resazurin assay. The molecular weights corresponding to the nominal low (10 kDa), medium (20 kDa), and high (40 kDa) polymers are given in Table 3 (n = 3). The three polymers in bold were explored in more detail as they showed good levels of antibacterial activity and low cytotoxicity.

Polymer Mn	Polymer Name	MICs for Different Target Microorganisms							
		*B. subtilis*		*E. coli*		*M. luteus*		*S. typhimurium*	
		µg/mL	µM	µg/mL	µM	µg/mL	µM	µg/ mL	µM
10 kDa	PDADMAC-10	16	1.4	32	2.7	32	2.7	64	5.4
17 kDa	**PDADMAC-20**	16	0.9	16	0.9	16	0.9	32	1.8
40 kDa	PDADMAC-40	16	0.4	16	0.4	16	0.4	32	0.9
12 kDa	PMETACL-10	64	6.2	64	6.2	64	6.2	64	6.2
18 kDa	PMETACL-20	64	3.7	64	3.7	64	3.7	64	3.7
37 kDa	**PMETACL-40**	32	0.9	32	0.9	32	0.9	64	1.7
14 kDa	PVMBT-10	64	4.7	64	4.7	64	4.7	128	9.3
21 kDa	PVMBT-20	32	1.5	32	1.5	64	3.1	64	3.1
37 kDa	**PVMBT-40**	32	0.9	32	10.9	32	0.9	64	1.7

**Table 5:** Selective toxicity of PDADMAC-20, PMETACL-40, and PVMBT-40.

Polymer	MIC (µg/mL)				HC_50_ mg/mL	Selectivity HC_50_/MIC
	*E. coli*	*B. subtilis*	*M. luteus*	*S.* *typhimurium*		
PDADMAC-20	16	16	16	32	>26	>800
PVMBT-40	32	32	32	64	>51	>800
PMETACL-40	32	32	32	64	>51	>800

## Data Availability

The data is contained in the manuscript or supplemantary materials.

## Supplementary Materials

The following supporting information can be downloaded at: https://www.mdpi.com/article/10.3390/antibiotics12081320/s1. Figure S1. (a) ^1^H NMR spectra of chain transfer agent 3 recorded in D_2_O, with peak assignments, (b) ^13^C NMR of the chain transfer agent 2 recorded in D_2_O, with peak assignments. Figure S2. ^1^H NMR spectra of poly(2-(dimethylamino)ethyl methacrylate) recorded in CDCl_3_, with peak assignments. E, D represent the peaks of the polymer’s main chain; A, B, C represent the peaks of the polymer’s side chain. Figure S3. ^1^H NMR spectra of poly([2-(methacryloyloxy)ethyl]trimethylammonium chloride) recorded in D_2_O, with peak assignments. E, D represent the peaks of the polymer’s main chain; A, B, C represent the peaks of the polymer’s side chain. Inset (F, G, H) shows the region of the RAFT agent. Figure S4. ^1^H NMR spectra of poly[3-(methacryloylamino)propyl]trimethylammonium chloride recorded in D_2_O, with peak assignments. E, D represent the peaks of the polymer’s main chain; A, B, C represent the peaks of the polymer’s side chain. Inset (F, G, H) shows the region of the RAFT agent. Figure S5. ^1^H NMR spectra of poly(vinylbenzyl trimethylammonium chloride) recorded in D_2_O, with peak assignments. E, D represent the peaks of the polymer’s main chain; A, B, C represent the peaks of the polymer’s side chain. Figure S6. ^1^H NMR spectra of poly(diallyldimethyl ammonium chloride) recorded in D_2_O, with peak assignments. C and D represent the peaks of the polymer’s main chain; A and B represent the peaks of the polymer’s side chain. Figure S7. Assessment of HeLa cell viability in the presence of five homopolymers (Mw ~20 kDa) using an MTT assay. Figure S8. GPC analysis of three different molecular weight of PDADMACs, PMETCLs, PVMBTs with elution using an aqueous buffer of 0.50 M acetic acid and 0.30 M NaH_2_PO_4_ (pH 2.5) at a flow rate of 1.0 mL min^−1^ at 25 °C. Peaks relative to poly(ethylene glycol) standards. Figure S9. Agar plates were inoculated with the target bacterial species, wells of 1 cm diameter were punched into the plates, and 100 µL of polymer solutions (at 2× MIC) were added into the wells and incubated overnight before the zones of inhibition were measured. Figure S10. Cytotoxicity of different molecular weights of the polymers (PDADMACs, PVMBTs, and PMEATCLs). Table S1. Zones of inhibition (agar well diffusion assay) in cm.

## Abbreviations

RAFT: Reversible Addition Fragmentation Chain Transfer Polymerization; SEM: Scanning Electron Microscopy; PDMAEMA: poly(2-(dimethylamino)ethyl methacrylate); PMETACL: poly([2-(methacryloyloxy)ethyl]trimethylammonium chloride); PDADMAC: poly(diallyl dimethyl ammonium chloride); PMET3: poly[3-(methacryloylamino)propyl]trimethylammonium chloride; PVMB: poly(vinyl benzyl trimethylammonium chloride); PI: propidium iodide.
