# Supplementary material for: Size-Controlled Ammonium-Based Homopolymers as Broad-Spectrum Antibacterials
Source: Antibiotics (Basel). 2023 Aug 16;12(8):1320. doi: 10.3390/antibiotics12081320 (PMC10452032; doi:10.3390/antibiotics12081320)
Supplement: Supplementary file 1 [file antibiotics-12-01320-s001.zip › antibiotics-2562444-supplementary.pdf]

Supplementary Materials

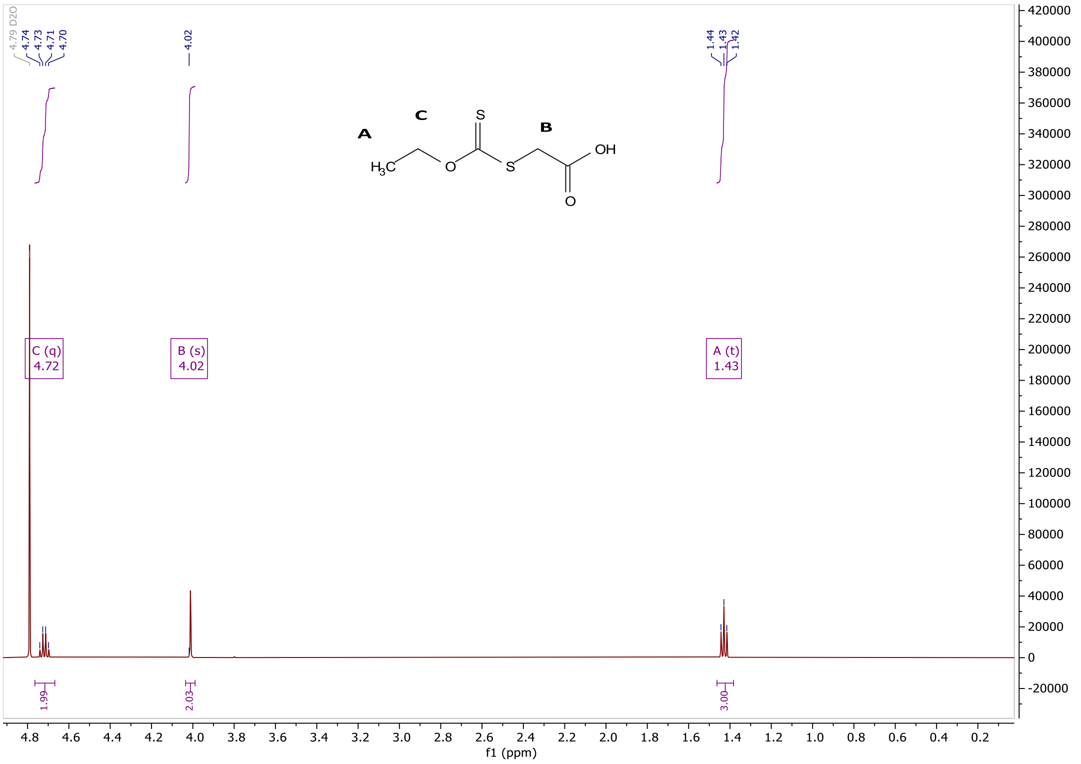

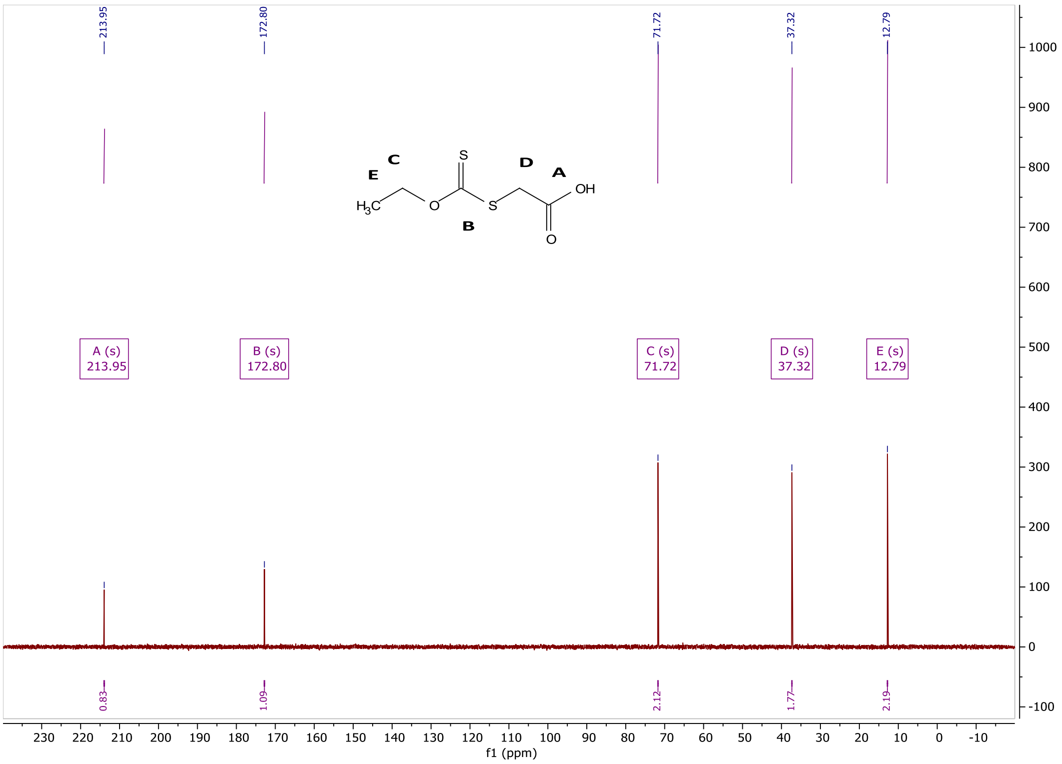

Figure S1. (a) ^{1}H NMR spectra of chain transfer agent 3 recorded in D_2O, with peak assignments, (b) ^{13}C NMR of the chain transfer agent 3 recorded in D_2O, with peak assignments.

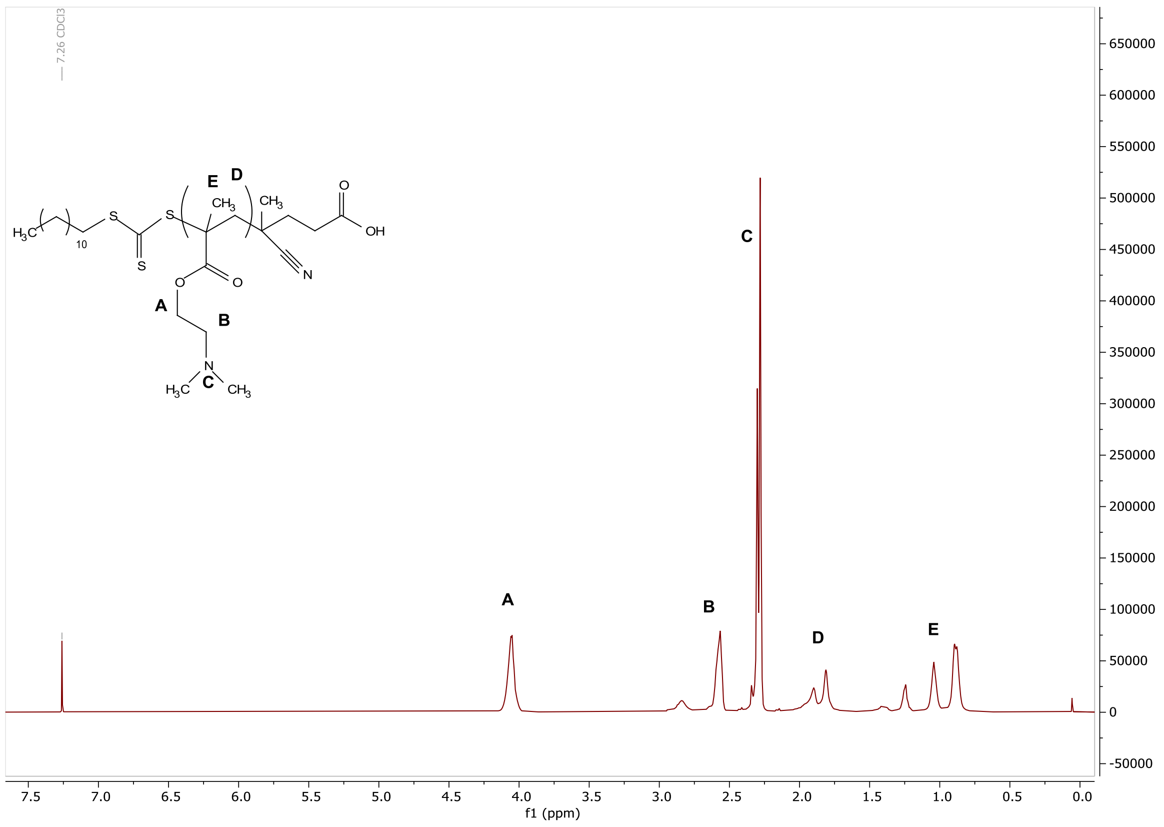

Figure S2. ^{1}H NMR spectra of poly(2-(dimethylamino)ethyl methacrylate) recorded in $CDCl_3$, with peak assignments. E, D represent the peaks of the polymer's main chain; A, B, C represent the peaks of polymer's side chain.

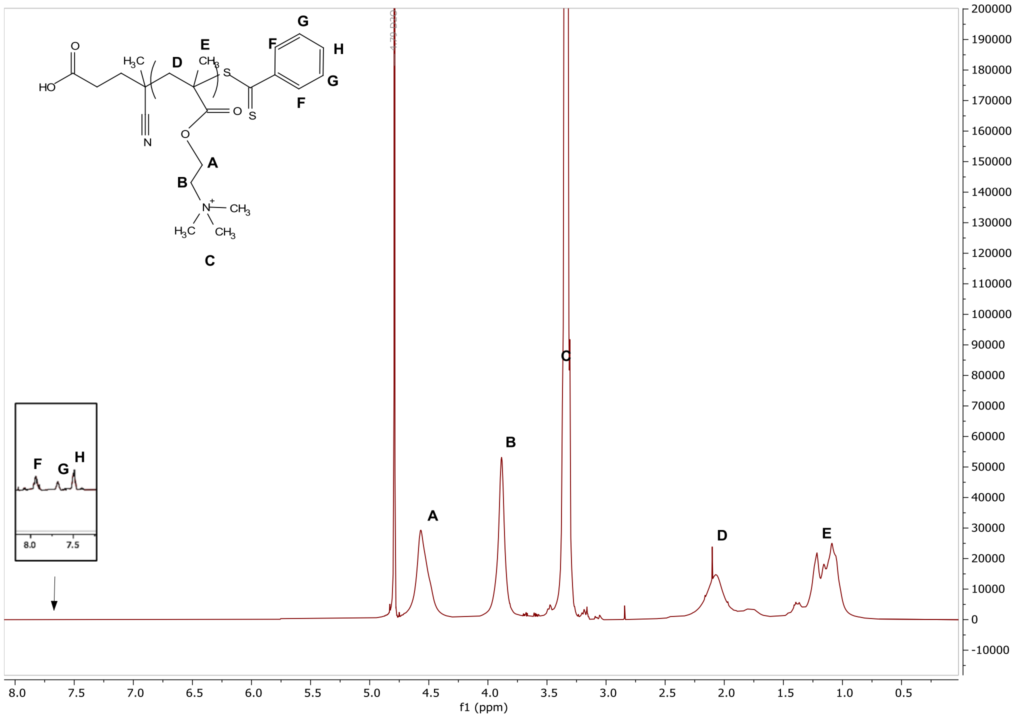

Figure S3. ^{1}H NMR spectra of poly([2-(methacryloyloxy)ethyl]trimethylammonium chloride) recorded in D_2O, with peak assignments. E, D represent the peaks of the polymer's main chain; A, B, C represent the peaks of polymer's side chain. Inset (F, G, H) shows the region of the RAFT agent.

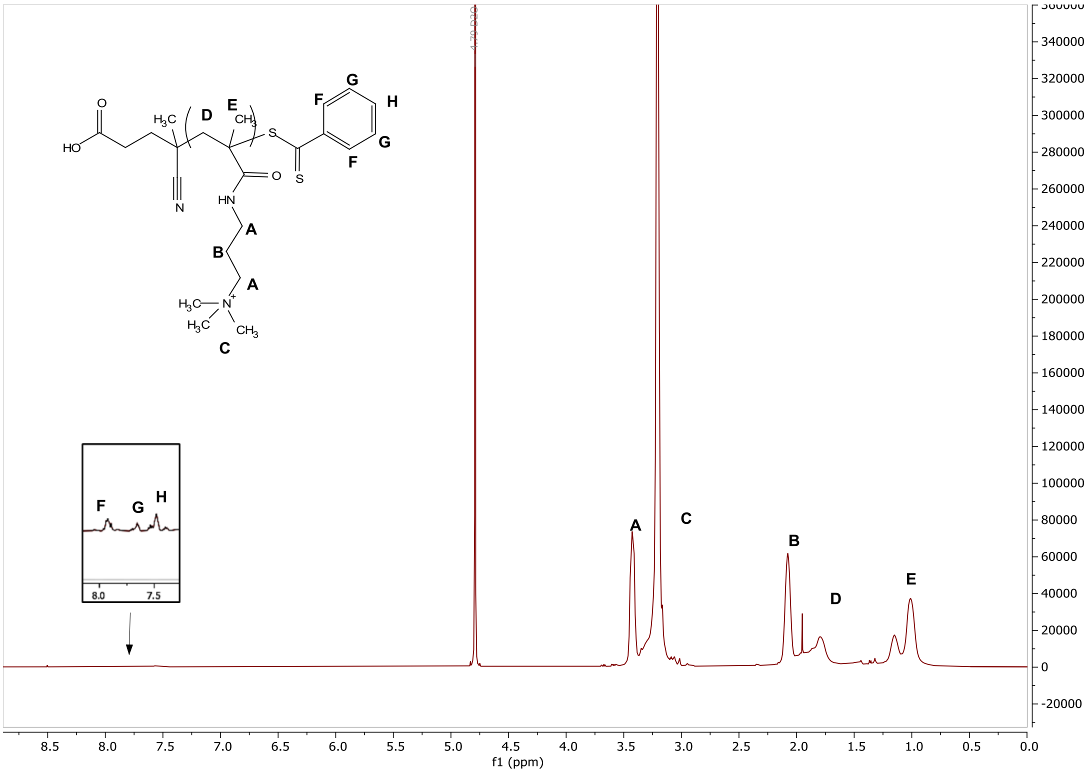

Figure S4. ^{1}H NMR spectra of poly[3-(methacryloylamino)propyl]trimethylammonium chloride recorded in D_2O, with peak assignments. E, D represent the peaks of polymer's main chain; A, B, C represent the peaks of polymer's side chain. Inset (F, G, H) shows the region of the RAFT agent.

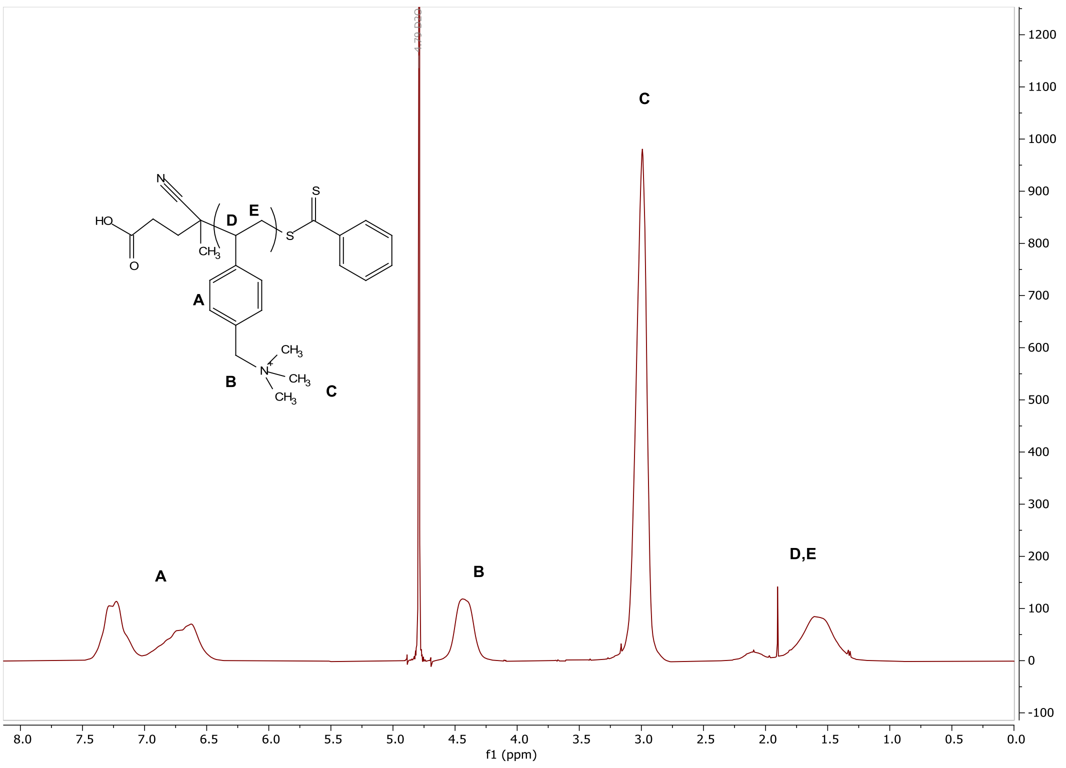

Figure S5. ^{1}H NMR spectra of poly(vinylbenzyl trimethylammonium chloride) recorded in D_2O, with peak assignments. E, D represent the peaks of polymer's main chain; A, B, C represent the peaks of polymer's side chain.

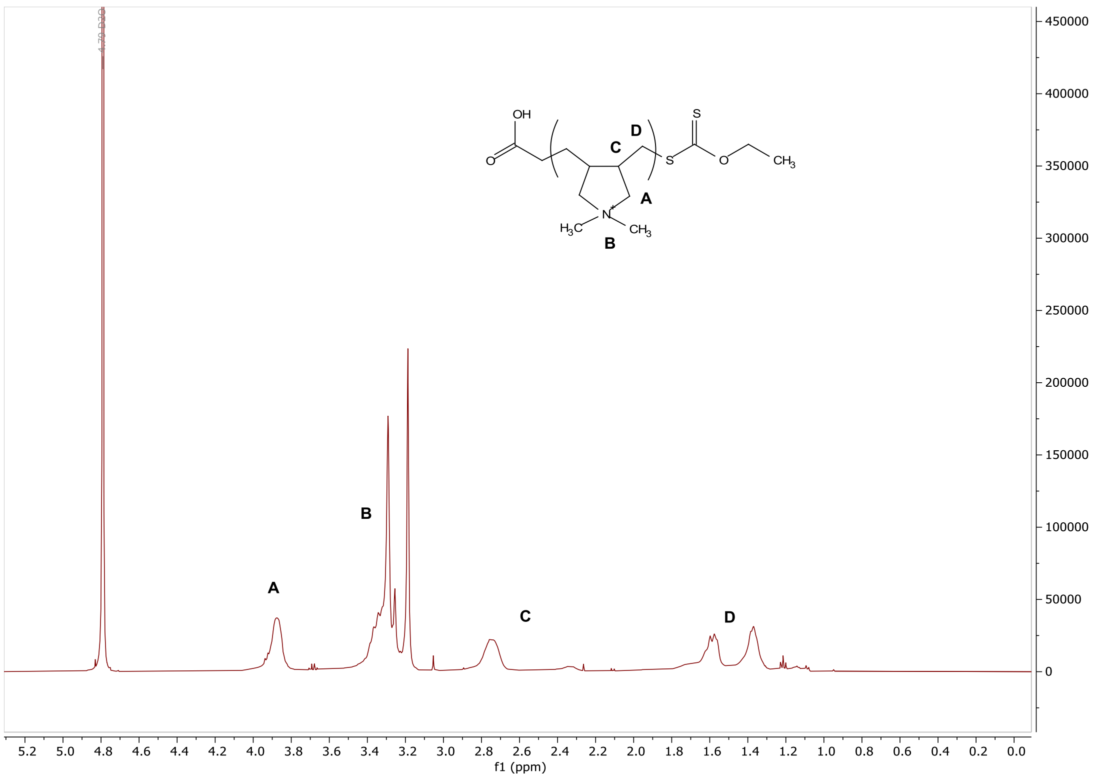

Figure S6. ^{1}H NMR spectra of poly (diallyldimethyl ammonium chloride) recorded in D_2O, with peak assignments. C and D represent the peaks of polymer's main chain; A and B represent the peaks of polymer's side chain.

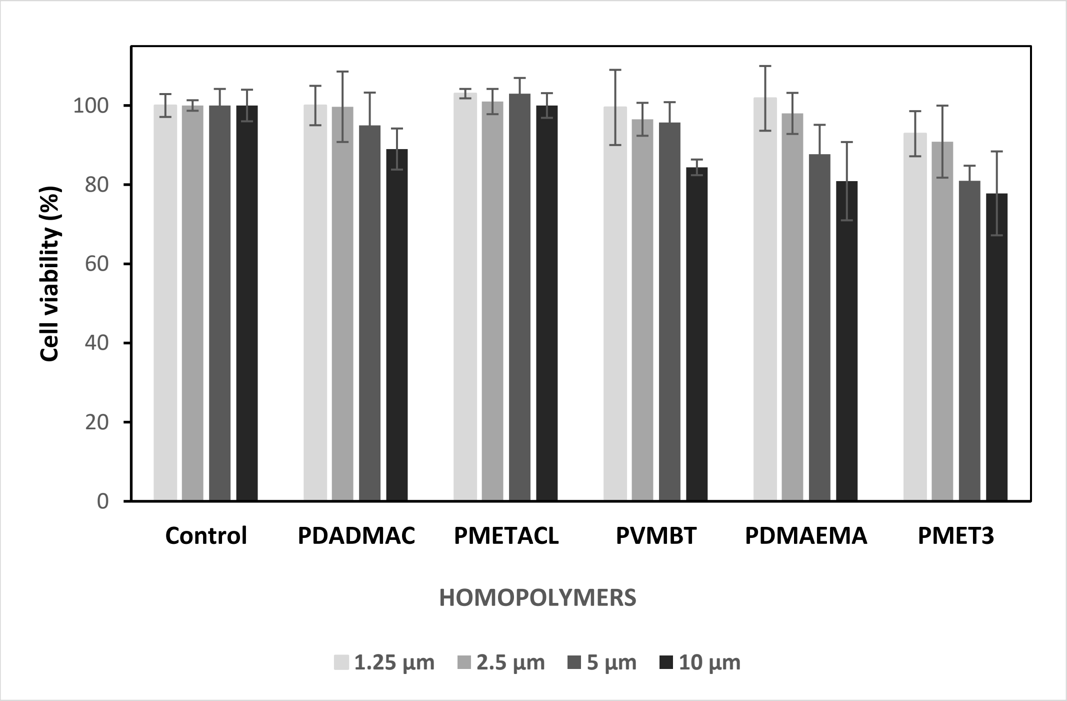

Figure S7. Assessment of HeLa cell viability in the presence of five homopolymers (Mw ~20 kDa) using an MTT assay.

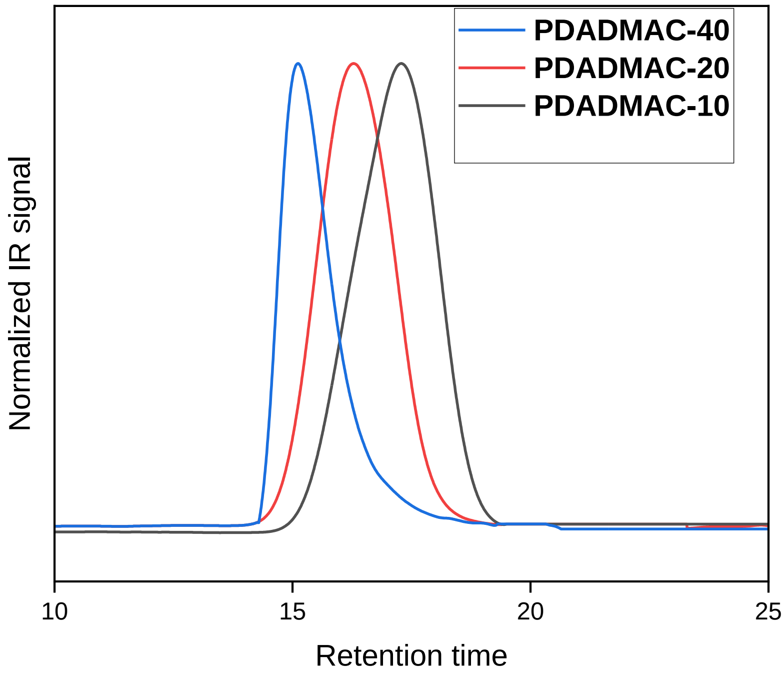

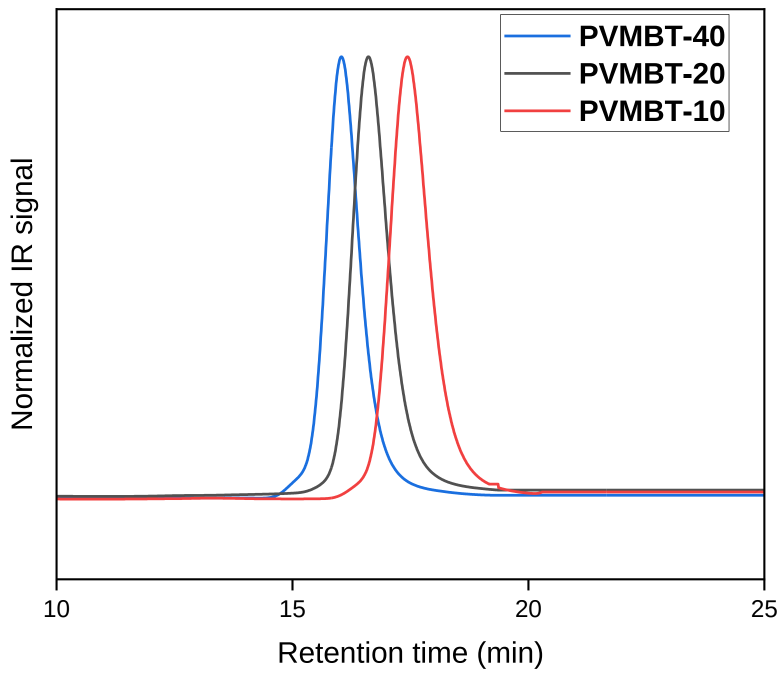

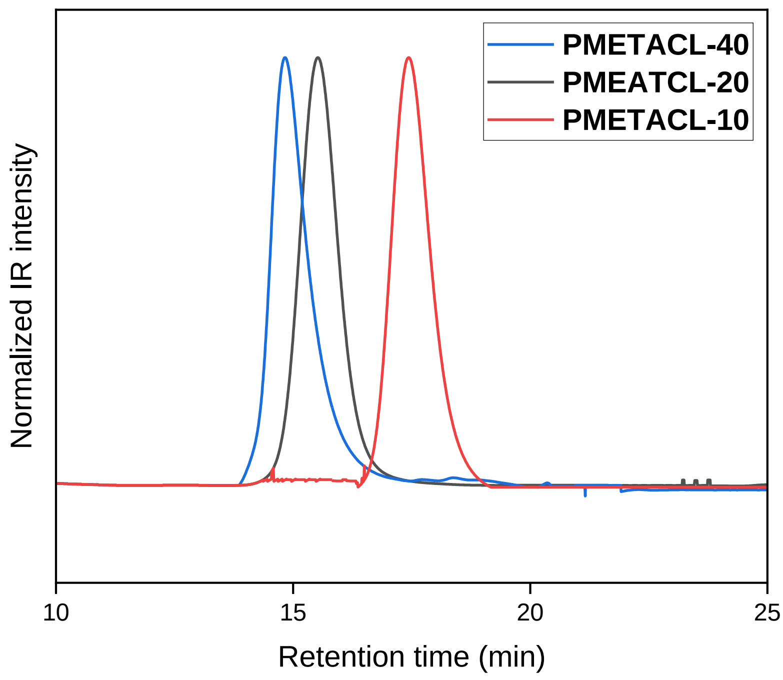

Figure S8. GPC analysis of three different molecular weight of PDADMACs, PMETCLs, PVMBTs with elution using an aqueous buffer of 0.50 M acetic acid and 0.30 M NaH_2PO_4 (pH 2.5) at a flow rate of 1.0 ml min^{-1} at 25 ˚C. Peaks relative to poly(ethylene glycol) standards.

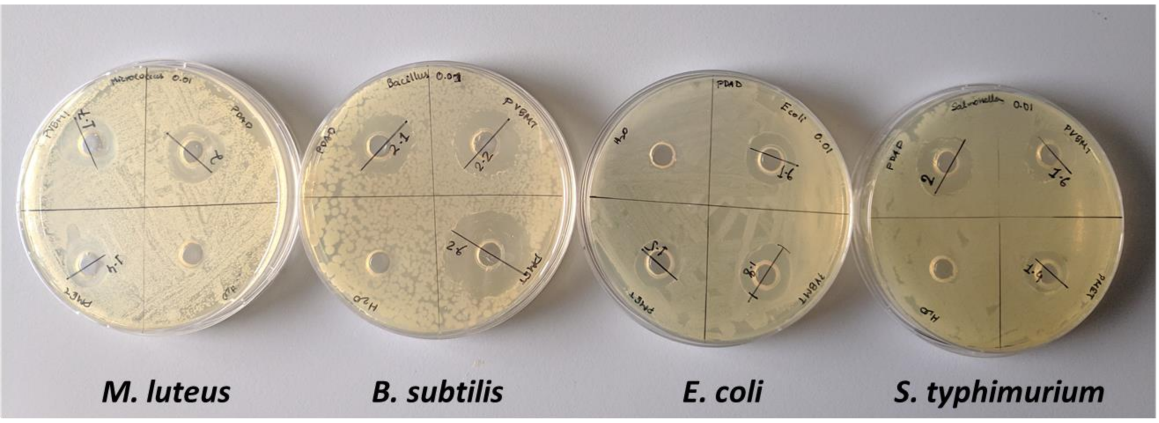

Figure S9. Agar plates were inoculated with the target bacterial species and wells of 1 cm diameter were punched into the plates and 100 µL of polymer solutions (at 2xMIC) were added into the wells and incubated overnight before the zones of inhibition were measured.

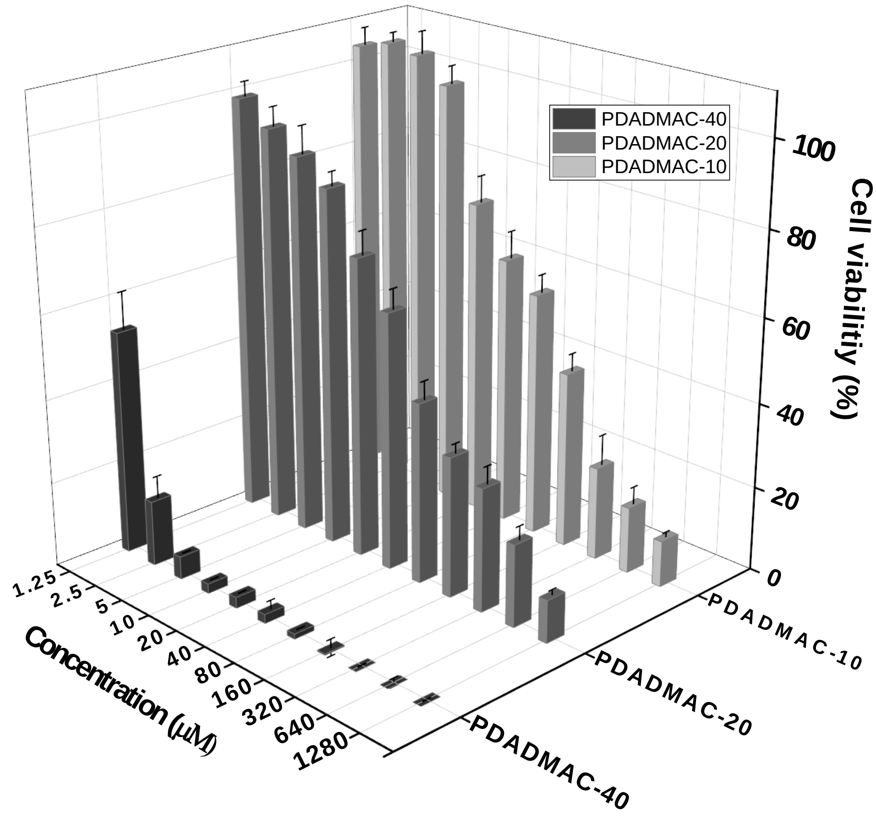

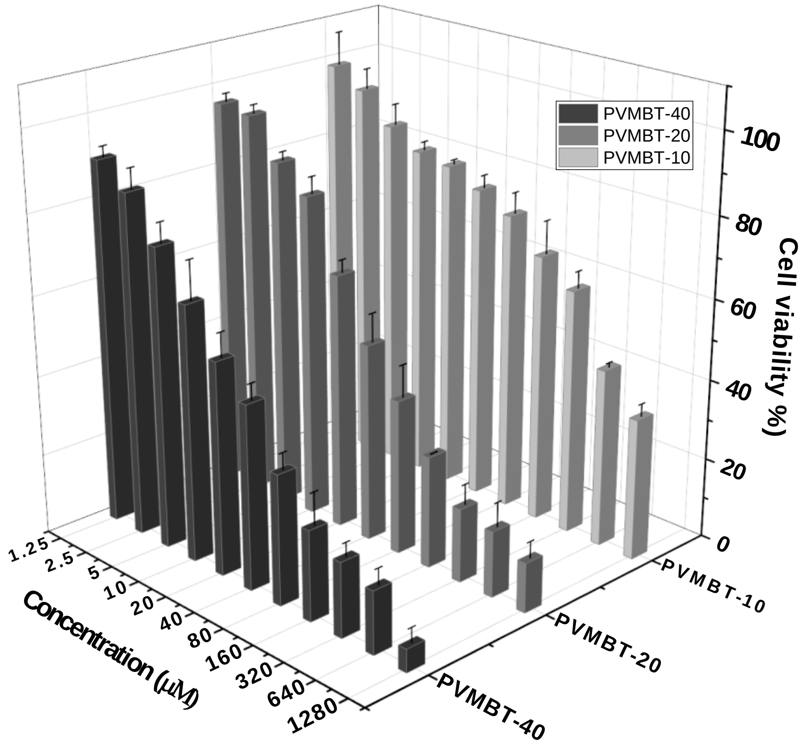

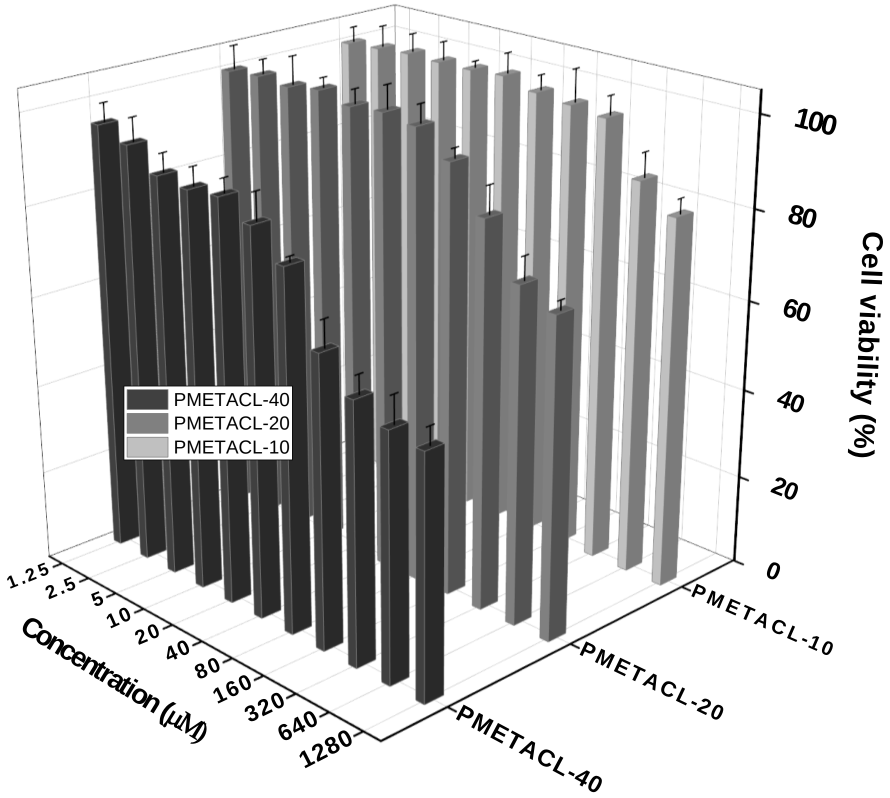

Figure S10. Cytotoxicity of different molecular weights of the polymers (PDADMACs, PVMBTs and PMEATCLs).

Table S1. Zone of inhibitions (agar well diffusion assay) in cm.

Polymers	***B. subtilis***	***M. luteus***	***E.coli***	***S. typhimurium***
PDADMAC-40	2.1	2	1.6	2.0
PMETACL-40	2.6	1.4	1.5	1.9
PVBMT-40	2.2	1.7	1.8	1.6
